# Characterization of Pathogenic *Photobacterium damselae* subsp. *damselae* From Whiteleg Shrimp (*Litopenaeus vannamei*): An Emerging Cause of Mass Mortality in the Republic of Korea

**DOI:** 10.1155/tbed/1555448

**Published:** 2026-08-06

**Authors:** Jae Hong Park, Sib Sankar Giri, Mae Hyun Hwang, Da Sol Park, Eun Jae Park, Sung Jae Han, Jin Woo Jun, Se Chang Park

**Affiliations:** ^1^ Laboratory of Aquatic Biomedicine, College of Veterinary Medicine and Research Institute for Veterinary Science, Seoul National University, Seoul 08826, Republic of Korea, snu.ac.kr; ^2^ Department of Food Science and Biotechnology, Gachon University, Seongnam 13120, Republic of Korea, gachon.ac.kr; ^3^ Department of Aquaculture, Korea National University of Agriculture and Fisheries, Jeonju 54874, Republic of Korea; ^4^ Startup Company SHA Bio Co., Ltd., College of Veterinary Medicine and Research Institute for Veterinary Science, Seoul National University, Seoul 08826, Republic of Korea, snu.ac.kr

**Keywords:** Korea, *Photobacterium damselae* subsp. *damselae*, shrimp disease, white muscle, whiteleg shrimp

## Abstract

Mass mortality events characterized by muscle whitening occurred in farmed whiteleg shrimp (*Litopenaeus vannamei*) in the Republic of Korea from July to September 2025. After ruling out the major crustacean pathogens listed by the World Organization for Animal Health (WOAH) through molecular screening, three dominant isolates of *Photobacterium damselae* subsp. *damselae* were recovered from the affected shrimp. The isolates were identified by 16S rRNA gene sequencing and confirmed by detection of the *ureC* gene. Phenotypic characterization showed that *P. damselae* subsp. *damselae* SNUABM‐JN1 grew across a wide range of temperatures (10–40°C) and salinities (0.5%–5.0%), with a shorter lag phase at 29°C across the tested salinity range. In the challenge test, the median lethal dose (LD_50_) was estimated as 1.10 × 10^5^ CFU⋅g^−1^ at 25°C and 5.58 × 10^3^ CFU⋅g^−1^ at 29°C, indicating lower survival at the higher temperature. The characteristic clinical signs of muscle whitening were reproduced, and histopathology revealed coagulative necrosis and hemocytic infiltration in both muscle and hepatopancreatic tissues. Whole genome sequencing showed that *P. damselae* subsp. *damselae* SNUABM‐JN1 possesses two chromosomes and four plasmids, with 4420 predicted genes. Genomic analysis identified 58 putative virulence–associated genes and seven antimicrobial resistance genes. In addition, six prophage regions were predicted, including one intact prophage carrying a ribosome‐associated toxin gene (*ratA*), and 24 genomic islands were detected. Collectively, this study isolated and characterized *P. damselae* subsp. *damselae* SNUABM‐JN1 from whiteleg shrimp with muscle whitening, reporting this infection in the Republic of Korea for the first time.

## 1. Introduction

Whiteleg shrimp (*Litopenaeus vannamei*) is an economically important aquaculture species. In 2022, the global aquaculture production of whiteleg shrimp reached 6.8 million tons, making it the most produced single aquaculture species by volume [1]. Major producing countries include China, India, Indonesia, Vietnam, and Thailand, and the Republic of Korea also contributes to global production, with a reported production of 9504 tons in 2022 [2]. Despite their economic importance, bacterial diseases continue to cause mass mortality in farmed shrimp and impose substantial economic losses on the aquaculture industry [3]. Disease risks are often amplified by environmental and management factors, including temperature, salinity, and stocking density, because these variables can simultaneously affect pathogen proliferation and host susceptibility [4]. Therefore, reducing the risk of bacterial diseases in whiteleg shrimp aquaculture requires an approach that identifies the etiological agents and clarifies their occurrence patterns within region‐specific farming systems. Given the ongoing increases in seawater temperatures due to global warming, it is important to consider temperature‐associated shifts in both pathogen virulence and host vulnerability when establishing disease prevention and control strategies [5].

*Photobacterium damselae* subsp. *damselae* is a gram‐negative, halophilic bacterium belonging to the family *Vibrionaceae*, which is widely distributed in marine environments [6]. It was originally described as *Vibrio damsela* and subsequently underwent multiple taxonomic revisions, ultimately classified as *P. damselae* with two subspecies, subsp. *damselae* and subsp. *piscicida* [7–10]. Unlike subsp. *piscicida*, a well‐known causative agent of pasteurellosis in marine fish, subsp. *damselae* exhibits a broader host range and is associated with diverse clinical manifestations [11, 12]. With increasing reports of infection in marine aquaculture and an expanding geographic range of reported cases, *P. damselae* subsp. *damselae* has been recognized as an emerging pathogen [13]. It has also been isolated from various wild marine animals [14–16]. In addition, it can act as an opportunistic human pathogen, causing severe outcomes such as sepsis and necrotizing fasciitis after wound exposure to seawater or contact with seafood [17]. In shrimp, infections caused by *P. damselae* subsp. *damselae* have been reported in multiple countries, including Japan, China, India, and Thailand, and involve genera such as *Litopenaeus*, *Marsupenaeus*, and *Exopalaemon*, highlighting their potential threat to shrimp aquaculture [18–21].

From July to September 2025, mass mortality occurred on whiteleg shrimp farms located near the west coast of the Republic of Korea, and the affected shrimp exhibited white muscle lesions. Molecular screening for major crustacean pathogens listed by the World Organization for Animal Health (WOAH) was negative, prompting bacterial isolation and identification of the causative agent. The isolated *P. damselae* subsp. *damselae* underwent phenotypic characterization and antimicrobial susceptibility testing, and the experimental challenge reproduced clinical features comparable to those observed in the field. Genomic characterization was conducted using whole genome sequencing. Collectively, this study identified and characterized pathogenic *P. damselae* subsp. *damselae* associated with muscle whitening in whiteleg shrimp, representing the first report on this association in the Republic of Korea. These findings are expected to provide a basis for future studies on *P. damselae* subsp. *damselae* infection and pathogenesis in shrimp and to support the development of prevention and control strategies.

## 2. Materials and Methods

### 2.1. Sample Collection and Molecular Detection of Major Shrimp Pathogens

Between July and September 2025, moribund whiteleg shrimp exhibiting whitish muscle discoloration were collected from shrimp farms in Chungcheongnam‐do, Jeollanam‐do, and Jeollabuk‐do in the Republic of Korea. The specimens were transported to the laboratory on ice, and the external surfaces were disinfected with 70% ethanol prior to processing.

To screen for major crustacean pathogens, molecular detection assays were performed in accordance with the WOAH diagnostic recommendations. The hepatopancreas, gills, and muscles of each shrimp were aseptically dissected and homogenized. Total nucleic acids were extracted using the Patho Gene‐spin DNA/RNA Extraction Kit (iNtRON Biotechnology, Seongnam, Republic of Korea) according to the manufacturer’s instructions. For DNA‐based detection, conventional PCR was conducted using WOAH‐recommended primer sets to test for acute hepatopancreatic necrosis disease (AHPND), decapod iridescent virus 1 (DIV1), *Hepatobacter penaei*, infectious hypodermal and hematopoietic necrosis virus (IHHNV), white spot syndrome virus (WSSV), and *Enterocytozoon hepatopenaei* (EHP) [22–27]. Detailed information on the primers, amplification conditions, and expected product sizes is presented in Table S1. For RNA virus detection, the extracted RNA was treated with DNase I (RNase‐free; Thermo Scientific, Vilnius, Lithuania) and reverse‐transcribed into cDNA using a Power cDNA Synthesis Kit (iNtRON Biotechnology, Seongnam, Republic of Korea) according to the manufacturer’s protocol. RT‐PCR assays were performed with WOAH‐recommended primer sets to detect infectious myonecrosis virus (IMNV), *Macrobrachium rosenbergii* nodavirus (MrNV), Taura syndrome virus (TSV), and yellow head virus genotype 1 (YHV1) [28–31]. The primers, thermal cycling conditions, and expected amplicon sizes used for the RT‐PCR assays are listed in Table S2. The PCR and RT‐PCR products were electrophoresed on a 1% agarose gel containing the StaySafe Nucleic Acid Reagent (Real Biotech, Banqiao, Taiwan) and confirmed using ultraviolet transillumination.

### 2.2. Bacterial Isolation, Identification, and Phylogenetic Analysis

The muscle and hepatopancreas were aseptically dissected from diseased shrimp and homogenized in 1 mL of phosphate‐buffered saline (PBS). The homogenates were serially diluted in PBS, and aliquots were spread‐plated onto thiosulfate citrate bile salts sucrose (TCBS) agar and tryptic soy agar supplemented with 1.5% NaCl (TSA+). The plates were incubated at 25°C for 24 h. Representative and predominant single colonies were subcultured on TSA + plates to obtain pure cultures.

Genomic DNA was extracted from the bacterial isolates using the DNeasy Blood and Tissue Kit (Qiagen, Hilden, Germany) according to the manufacturer’s instructions. The 16S rRNA gene was amplified with the universal primers 27F and 1492R, and the PCR products were sequenced by Macrogen (Seoul, Republic of Korea) with universal primers 785F and 907R. The resulting sequences were compared to the NCBI database using BLAST to identify the isolates (http://blast.ncbi.nlm.nih.gov/).

For phylogenetic analysis, the 16S rRNA gene sequences of 20 *P. damselae* subsp. *damselae* strains and nine additional *Photobacterium* species were retrieved from GenBank and analyzed together with the sequences obtained in this study. Sequences were aligned using the MUSCLE program implemented in MEGA software (v12.0.11), and a maximum likelihood phylogenetic tree was constructed with 1000 bootstrap replicates [32].

To further discriminate the subspecies of the isolates, PCR was performed targeting the *ureC* gene, which is present in *P. damselae* subsp. *damselae* but absent in *P. damselae* subsp. *piscicida* [33]. The *ureC* gene was amplified using the primer pair *ureC*‐F (5′‐TCCGGAATAGGTAAAGCGGG‐3′) and *ureC*‐R (5′‐CTTGAATATCCATCTCATCTGC‐3′). PCR was carried out with an initial denaturation at 95°C for 4 min, followed by 30 cycles of denaturation at 95°C for 1 min, annealing at 60°C for 1 min, and extension at 72°C for 40 s, with a final extension at 72°C for 5 min. The PCR products were electrophoresed on a 1% agarose gel containing StaySafe Nucleic Acid Reagent and examined under ultraviolet transillumination. Gel images were visualized using a GelDoc Go system (Bio‐Rad, CA, USA).

### 2.3. Morphological and Phenotypic Characterization of the Pathogen

Colony morphology of *P. damselae* subsp. *damselae* SNUABM‐JN1 was evaluated after aerobic incubation on TSA+ and TCBS agar at 25°C for 24 h. Colony color, shape, elevation, and margin were recorded on each medium. Gram staining was performed using a Color Gram 2 kit (bioMérieux, Marcy l’Étoile, France) according to the manufacturer’s instructions. Cell morphology was further examined by transmission electron microscopy (TEM). Bacterial cells were washed with PBS, negatively stained with 2% (w/v) uranyl acetate for 15 s, and examined using a JEM‐2100Plus transmission electron microscope (JEOL Ltd., Tokyo, Japan) operated at 200 kV.

To determine the growth temperature range of *P. damselae* subsp. *damselae* SNUABM‐JN1, bacterial cultures were inoculated onto TSA+ plates and incubated at 5, 10, 15, 20, 25, 30, 35, 40, 45, and 50°C for 72 h. Motility was evaluated using a previously described method [34]. For the swimming assay, an overnight culture of SNUABM‐JN1 was inoculated into the center of tryptic soy broth (TSB) supplemented with 1.5% NaCl (TSB+) plates containing 0.2% agar, using a sterile needle. Plates were incubated at 25°C, and the diameters of the swimming zones were measured at 6 and 12 h. For the swarming assay, 2 µL of bacterial suspension was spotted onto TSB+ plates containing 0.5% agar and incubated at 25°C. The diameters of the swarming zones were measured at 12 and 36 h. Biochemical profiling was performed using the VITEK 2 system with a VITEK 2 GN identification card (bioMérieux, Marcy l’Etoile, France) according to the manufacturer’s instructions.

To assess the NaCl tolerance of strain SNUABM‐JN1, overnight cultures were incubated at 25°C or 29°C and then inoculated at 1% (v/v) into TSB adjusted to final NaCl concentrations of 0.5%, 1.0%, 1.5%, 2.0%, 2.5%, 3.0%, 3.5%, 4.0%, 4.5%, and 5.0%. Each broth culture was incubated at the same temperature used for the overnight culture, and the optical density at 600 nm (OD_600_) was measured hourly for 24 h. All assays were performed in triplicate.

### 2.4. Antimicrobial Susceptibility Test

The antimicrobial susceptibility of *P. damselae* subsp. *damselae* SNUABM‐JN1 to 14 antimicrobial agents was evaluated using the disk diffusion method. A bacterial suspension was adjusted to a 0.5 McFarland standard and inoculated onto Mueller Hinton agar (MHA) using a sterile cotton swab to obtain a bacterial lawn. Antimicrobial susceptibility test disks (Oxoid, NY, USA) were placed on the agar surface, and plates were incubated at 25°C for 24 h. After incubation, the diameters of the inhibition zones were measured and interpreted as susceptible (S), intermediate (I), or resistant (R) according to the Clinical and Laboratory Standards Institute (CLSI) guidelines M45 for *Vibrio* spp. [35]. *Escherichia coli* ATCC 25922 was used as the quality control strain, and all assays were performed in triplicate.

### 2.5. Experimental Challenge Test

A total of 500 healthy whiteleg shrimp were obtained from a commercial shrimp farm in Gochang, Republic of Korea, and transported to the laboratory. The mean body weight was 12.05 ± 0.24 g. Shrimp were divided equally into two temperature groups and acclimated for 7 days in continuously aerated filtered artificial seawater adjusted to 2% salinity at either 25°C or 29°C. Prior to the bacterial challenge, several shrimp were randomly selected and screened by PCR and RT‐PCR for major crustacean pathogens using hepatopancreas, gill, and muscle tissues.

For experimental infection, *P. damselae* subsp. *damselae* SNUABM‐JN1 was cultured at the corresponding experimental temperature and prepared for intramuscular injection. Each shrimp was injected with 20 µL of the bacterial suspension at doses of 1.72 × 10^2^, 1.72 × 10^3^, 1.72 × 10^4^, 1.72 × 10^5^, 1.72 × 10^6^, or 1.72 × 10^7^ CFU⋅g^−1^ into the abdominal muscle at the junction between the third and fourth abdominal segments. Control shrimps received equal volumes of PBS. Shrimp were maintained at a density of 10 individuals per tank containing 50 L of continuously aerated artificial seawater at 2% salinity. Each dose group was tested in triplicate tanks. Water temperature was maintained at 25 ± 0.5°C or 29 ± 0.5°C depending on the acclimation group, and a daily 50% water exchange was performed using artificial seawater adjusted to the same temperature and salinity. Shrimps were monitored for up to 30 days postinjection (dpi). Dead shrimp were removed immediately, and bacterial reisolation was performed to confirm whether the mortality was attributable to the injected strain.

For histopathological examination, muscle and hepatopancreatic tissues were collected from the infected shrimp and fixed in Davidson’s AFA solution. Fixed tissues were dehydrated using an ethanol series, embedded in paraffin, sectioned, and stained with hematoxylin and eosin. The slides were examined under a light microscope and digitally scanned using a Pannoramic 250 Flash III scanner (3D HISTECH Ltd., Budapest, Hungary).

### 2.6. Whole Genome Sequencing and Analysis

The genomic DNA of *P. damselae* subsp. *damselae* SNUABM‐JN1 was sequenced using a hybrid approach that combined the Illumina NovaSeq X system (Illumina, CA, USA) and the PacBio Revio system (Pacific Biosciences, CA, USA). The Illumina short‐read library was prepared using the TruSeq Nano DNA kit (Illumina, CA, USA), and the PacBio long‐read library was prepared using the SMRTbell prep kit 3.0 (Pacific Biosciences, CA, USA). Raw Illumina reads were assessed for quality using FastQC (v0.11.7), followed by adapter trimming and quality filtering using Trimmomatic (v0.38). Reads were retained only if at least 90% of the bases had a Phred quality score of 30 or higher. De novo assembly was performed using PacBio HiFi reads in Hifiasm (v0.19.9). The assembly was polished using Inspector (v1.0.1), and additional error correction was conducted using Pilon (v1.22). Assembly completeness was evaluated using BUSCO (v5.1.3).

Gene prediction and functional annotation were performed using Prokka (v1.14.6), eggNOG‐mapper (v2.1.10, eggNOG database v5.0.2), InterProScan (v5.34‐73.0), and Kyoto Encyclopedia of Genes and Genomes (KEGG) [36]. Putative virulence genes and antimicrobial resistance genes were identified using the Virulence Factor Database (VFDB) [37] and the Comprehensive Antibiotic Resistance Database (CARD) [38]. Prophage regions were predicted using PHASTER [39], and genomic islands were predicted using IslandViewer 4 [40].

### 2.7. Statistical Analysis

Statistical analyses were performed using GraphPad Prism 9.0 (GraphPad Software, CA, USA). Data are presented as the mean ± standard deviation (SD) of triplicate experiments. Survival data from the challenge test were analyzed using the Kaplan–Meier method, and survival curves were compared using the log‐rank (Mantel–Cox) test. For each temperature condition, the overall difference among inoculum dose groups was evaluated, followed by pairwise comparisons between the control and each dose group, with *p*‐values adjusted using the Bonferroni correction. Survival was also compared between the two temperature conditions at matched inoculum doses using the log‐rank test. *p*  < 0.05 was considered statistically significant.

## 3. Results

### 3.1. Isolation and Identification of *P. damselae* subsp. *damselae*

No major crustacean pathogens listed by WOAH were detected in the diseased shrimp by PCR or RT‐PCR. From the dominant bacterial colonies isolated from the whitish muscle tissue and hepatopancreas, three representative isolates were selected and designated SNUABM‐JN1, SNUABM‐JB1, and SNUABM‐CN1.

**Figure 1:**
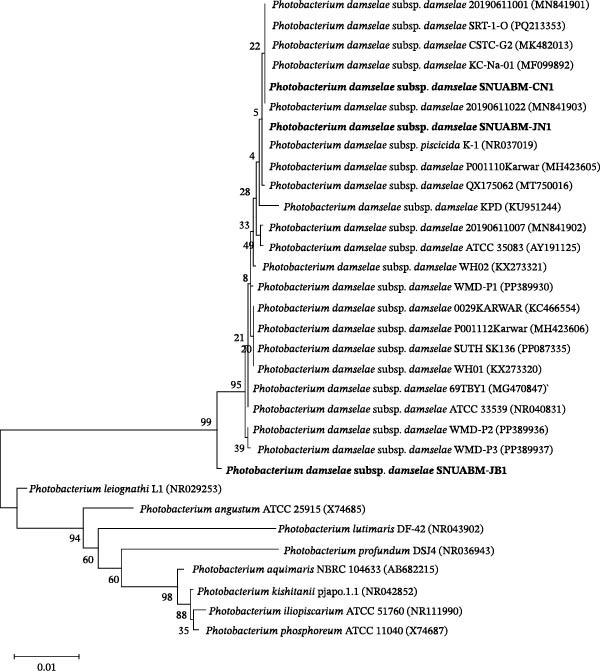
Maximum likelihood phylogenetic tree based on 16S rRNA gene sequences of three isolates obtained in this study. The analysis included 20 *Photobacterium damselae* subsp. *damselae* strains and nine additional *Photobacterium* species retrieved from GenBank. The isolates obtained in this study are shown in bold. The phylogenetic tree was constructed using MEGA software (v12.0.11) with 1000 bootstrap replicates.

Based on 16S rRNA gene sequence comparisons against the NCBI database, the closest matches for SNUABM‐JN1, SNUABM‐JB1, and SNUABM‐CN1 were *P. damselae* subsp. *damselae* KC‐Lo52‐R1 (MN192090; 99.86%), *P. damselae* subsp. *damselae* KC‐Na26‐B2 (MN263230; 99.51%), and *P. damselae* subsp. *damselae* SUTH_T11L (PP087317; 100%), respectively. In a maximum likelihood phylogenetic tree constructed using 16S rRNA gene sequences from the three isolates, together with type strains of *Photobacterium* spp. and additional *P. damselae* subsp. *damselae* strains, all isolates clustered within the *P. damselae* clade (Figure 1). PCR targeting the *ureC* gene was performed to discriminate subspecies within *P. damselae*. All three isolates tested positive, confirming their identification as *P. damselae* subsp. *damselae* (Figure S1).

### 3.2. Morphological and Phenotypic Characteristics of *P. damselae* subsp. *damselae* SNUABM‐JN1

Morphological examination showed that the isolate formed cream‐colored, circular, convex colonies with entire margins on TSA+ (Figure S2A) and green, circular, convex colonies with entire margins on TCBS agar (Figure S2B). Gram staining revealed that the isolate was gram‐negative and exhibited a rod‐shaped morphology (Figure S2C). TEM analysis further confirmed the rod‐shaped morphology, with cells measuring 2.37 ± 0.15 µm in length and 0.93 ± 0.03 µm in width (*n* = 3), and revealed the presence of a single polar flagellum (Figure S2D).

The growth of *P. damselae* subsp. *damselae* SNUABM‐JN1 was observed at 10–40°C (Table 1). This strain exhibited swimming motility but did not display swarming motility. In the biochemical profiling, SNUABM‐JN1 was positive for L‐pyrrolydonyl‐arylamidase, β‐N‐acetyl‐glucosaminidase, D‐glucose, glucose fermentation, D‐maltose, D‐mannose, lysine decarboxylase, and coumarate.

**Table 1:** Phenotypic characteristics of the isolate.

Characteristics	Result	Characteristics	Result
Temperature range (°C)	10–40	Swimming	+
Swarming	−	Ala‐Phe‐Pro‐arylamidase	−
Adonitol	−	L‐pyrrolydonyl‐arylamidase	+
L‐arabitol	−	D‐cellobiose	−
β‐Galactosidase	−	H2S production	−
β‐N‐acetyl‐glucosaminidase	+	Glutamyl arylamidase pNA	−
D‐glucose	+	γ‐Glutamyl‐transferase	−
Fermentation of glucose	+	β‐Glucosidase	−
D‐maltose	+	D‐mannitol	−
D‐mannose	+	β‐Xylosidase	−
β‐Alanine arylamidase pNA	−	L‐proline arylamidase	−
Lipase	−	Palatinose	−
Tyrosine arylamidase	−	Urease	−
D‐sorbitol	−	Saccharose/sucrose	−
D‐tagatose	−	D‐trehalose	−
Citrate (sodium)	−	Malonate	−
5‐Keto‐D‐gluconate	−	L‐lactate alkalinization	−
α‐Glucosidase	−	Succinate alkalinization	−
β‐N‐acetyl‐galactosaminidase	−	α‐Galactosidase	−
Phosphatase	−	Glycine arylamidase	−
Ornithine decarboxylase	−	Lysine decarboxylase	+
L‐histidine assimilation	−	Coumarate	+
β‐Glucuronidase	−	O/129 resistance	−
Glu‐Gly‐Arg‐arylamidase	−	L‐malate assimilation	−
Ellman’s reagent	−	L‐lactate assimilation	−

*Note:* +, positive; −, negative.

**Figure 2:**
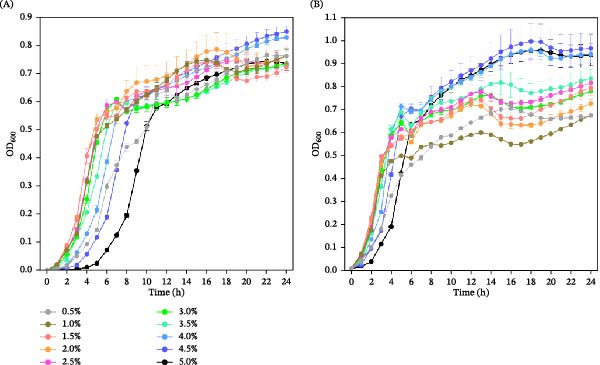
Growth curves of *P. damselae* subsp. *damselae* SNUABM‐JN1 under different NaCl concentrations at 25°C (A) and 29°C (B). Results are expressed as mean ± standard deviation of triplicate experiments.

Strain SNUABM‐JN1 generated growth curves across the full NaCl range tested (0.5%–5.0%) at both 25°C and 29°C. At 25°C, the lag phase was relatively short at 1.0%–3.5% NaCl, whereas an extended lag phase was observed under the lowest‐salinity condition (0.5% NaCl) and at higher salinities (4.0%–5.0% NaCl), with the longest lag phase at 5.0% NaCl (Figure 2A). The growth rate was the highest at 2.0% NaCl and the lowest at 5.0% NaCl. At 29°C, the lag phase was shortened across all salinities compared with 25°C (Figure 2B). Consistent with the 25°C condition, the longest lag phase was observed at 5.0% NaCl, whereas the highest growth rate occurred at 1.5% NaCl and the lowest at 0.5% NaCl.

### 3.3. Antimicrobial Susceptibility of the Pathogen

Antimicrobial susceptibility tests showed that *P. damselae* subsp. *damselae* SNUABM‐JN1 was susceptible to 14 antimicrobial agents, including ampicillin, ampicillin–sulbactam, cefepime, cefotaxime, cefoxitin, ceftazidime, imipenem, meropenem, amikacin, gentamicin, tetracycline, ciprofloxacin, ofloxacin, and chloramphenicol (Table S3).

### 3.4. Experimental Challenge Test of Whiteleg Shrimp With *P. damselae* subsp. *damselae* SNUABM‐JN1

**Figure 3:**
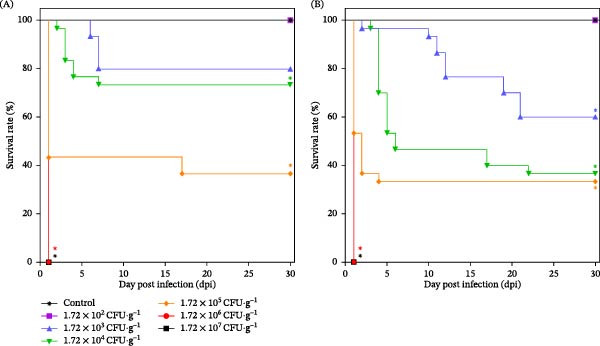
Kaplan–Meier survival curves of whiteleg shrimp following intramuscular challenge with *P. damselae* subsp. *damselae* SNUABM‐JN1 at 25°C (A) and 29°C (B). Each group consisted of three replicate tanks (*n* = 10 per tank). Asterisks indicate significant differences from the control group (log‐rank test with Bonferroni correction, *p*  < 0.05).

**Figure 4:**
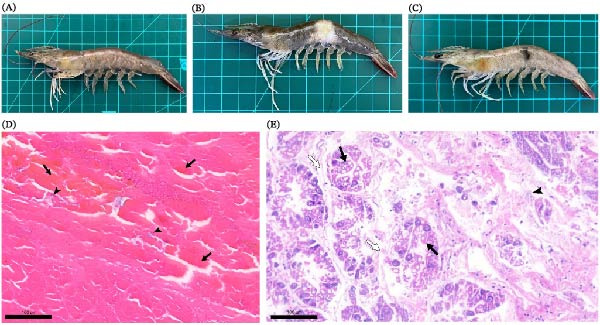
Clinical signs and histopathological changes in whiteleg shrimp following experimental infection with *P. damselae* subsp. *damselae* SNUABM‐JN1. (A) Control shrimp injected intramuscularly with PBS. (B) Representative gross appearance of whiteleg shrimp at 1 day postinfection (dpi). (C) Whiteleg shrimp exhibiting clinical signs at the late phase of infection. (D) Muscle tissue showing diffuse coagulative necrosis characterized by hypereosinophilia, nuclear loss, and hyalinization of muscle fibers (black arrows), accompanied by hemocytic infiltration (arrowheads). (E) Hepatopancreas revealing diffuse coagulative necrosis of hepatopancreatic tubules with epithelial sloughing (black arrows) and detachment from the surrounding connective tissue (open arrows). Hemocytic infiltration is also observed (arrowhead). Scale bars = 100 μm.

No major crustacean pathogens listed by the WOAH were detected by PCR or RT‐PCR in the shrimp used in the challenge experiments. Under each temperature condition, survival differed significantly among the inoculum dose groups (*p* < 0.0001). At 25°C, the final survival rates at 30 dpi were 80.00% in the 1.72 × 10^3^ CFU⋅g^−1^ group and 73.33% in the 1.72 × 10^4^ CFU⋅g^−1^ group (Figure 3A). In contrast, under the 29°C condition, the final survival rate decreased to 60.00% in the 1.72 × 10^3^ CFU⋅g^−1^ group and to 36.67% in the 1.72 × 10^4^ CFU⋅g^−1^ group, indicating a temperature‐related increase in mortality at the same inoculum doses (Figure 3B). In both temperature groups, shrimp injected with 1.72 × 10^6^ or 1.72 × 10^7^ CFU⋅g^−1^ reached 100% mortality within 1 dpi. No mortality or clinical signs were observed in the control group injected with PBS. Compared with the control, a significant reduction in survival was observed from 1.72 × 10^4^ CFU⋅g^−1^ at 25°C, whereas at 29°C the reduction became significant from a lower dose of 1.72 × 10^3^ CFU⋅g^−1^ (*p* < 0.05). Direct comparison between the two temperatures at matched doses showed the largest difference in survival at 1.72 × 10^4^ CFU⋅g^−1^, where survival was lower at 29°C than at 25°C. The median lethal dose (LD_50_) estimated using the LD50 calculator (Quest Graph, AAT Bioquest, Inc.) was 1.10 × 10^5^ CFU⋅g^−1^ at 25°C and 5.58 × 10^3^ CFU⋅g^−1^ at 29°C, showing that a lower bacterial dose was required to reach LD_50_ at 29°C. Compared with the PBS‐injected control (Figure 4A), the infected shrimp initially developed muscle whitening around the injection site, beginning at 1 dpi (Figure 4B). As the disease progressed, the whitening extended throughout the abdominal muscles (Figure 4C). Histopathological examination revealed diffuse coagulative necrosis and hemocytic infiltration of the muscle tissue (Figure 4D). The necrotic lesions were characterized by hypereosinophilia, nuclear loss, and hyalinization of muscle fibers. In the hepatopancreas, the histopathological changes included diffuse coagulative necrosis of the hepatopancreatic tubules with epithelial sloughing and detachment from the surrounding connective tissue and hemocytic infiltration (Figure 4E).

### 3.5. Genomic Characteristics of *P. damselae* subsp. *damselae* SNUABM‐JN1

**Figure 5:**
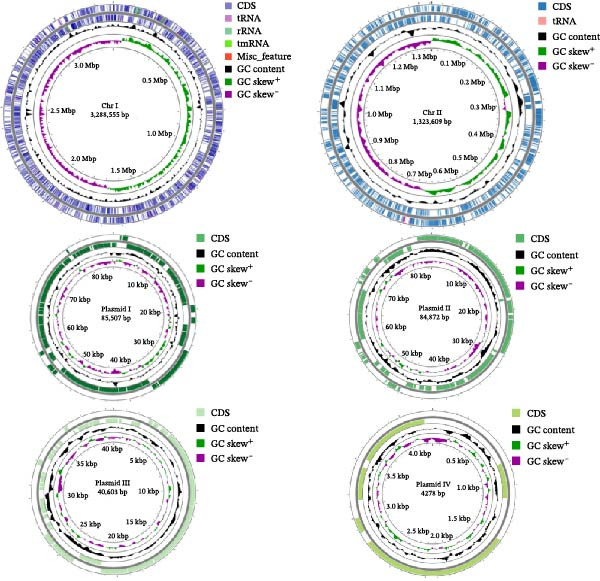
Circular genome maps of *P. damselae* subsp. *damselae* SNUABM‐JN1.

The final assembly of *P. damselae* subsp. *damselae* strain SNUABM‐JN1 comprised two circular chromosomes and four circular plasmids, and the assembly completeness assessed by BUSCO was 100% (Figure 5). Chromosome I (GenBank accession number CM132636) was 3,288,555 bp in length with a GC content of 41.5%, and Chromosome II (GenBank accession number CM132637) was 1,323,609 bp long, with a GC content of 39.1%. The four plasmids were 85,507 bp, 84,872 bp, 40,603 bp, and 4278 bp in length with GC contents of 39.9%, 42.8%, 37.7%, and 35.1%, respectively (GenBank accession numbers JBSPTP010000003–JBSPTP010000006). In total, 4420 genes were predicted, including 4158 protein‐coding sequences. The genome contained 208 tRNA and 53 rRNA genes, comprising 19 5S rRNAs, 17 16S rRNAs, and 17 23S rRNAs.

**Figure 6:**
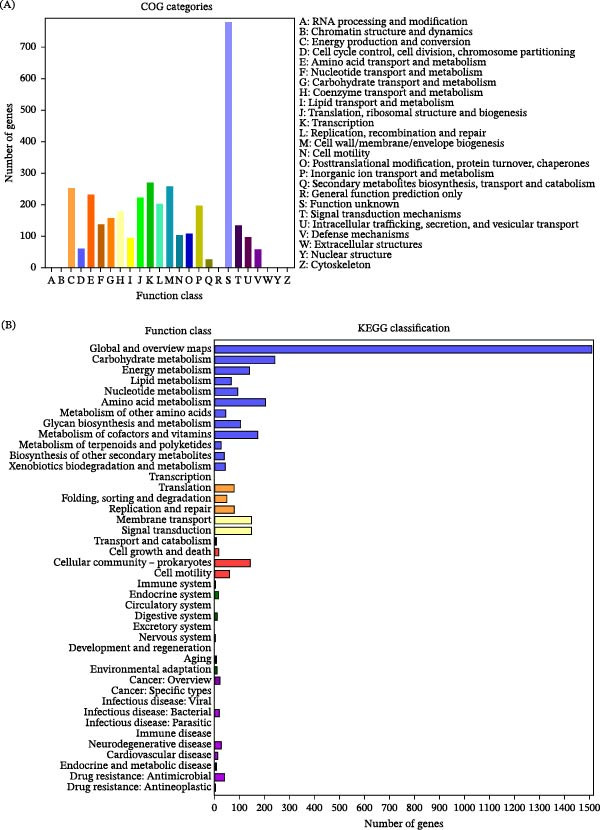
Functional annotation of *P. damselae* subsp. *damselae* SNUABM‐JN1. (A) Distribution of predicted protein‐coding genes assigned to Clusters of Orthologous Groups (COG) functional categories. (B) Kyoto Encyclopedia of Genes and Genomes (KEGG) functional classification of annotated genes grouped into six pathway categories—metabolism (blue), genetic information processing (orange), environmental information processing (yellow), cellular processes (red), organismal systems (green), and human diseases (purple).

Cluster of Orthologous Groups (COG) functional categories were assigned to 3568 protein‐coding genes (85.81%), and 3622 protein‐coding genes (87.11%) were mapped to KEGG pathways. The COG classification grouped 3568 genes into 20 functional classes, with transcription (7.57%); cell wall, membrane, and envelope biogenesis (7.23%); and energy production and conversion (7.06%) as the three most abundant categories (Figure 6A). KEGG annotation mapped 3622 genes to 42 categories, dominated by metabolism (74.38%), environmental information processing (7.21%), and cellular processes (6.35%) (Figure 6B).

Screening against the VFDB identified 58 putative virulence–associated genes related to adherence, antimicrobial activity and competitive advantage, biofilm formation, effector delivery systems, immune modulation, motility, nutritional and metabolic factors, regulation, and stress survival (Table S4). CARD analysis identified seven antimicrobial resistance genes, representing the following AMR gene families—elfamycin‐resistant EF‐Tu, penicillin‐binding protein mutations conferring resistance to β‐lactam antibiotics, the resistance‐nodulation‐cell division antibiotic efflux pump, and the small multidrug resistance antibiotic efflux pump (Table S5). The predicted resistance mechanisms include antibiotic efflux and alterations in antibiotic targets.

PHASTER identified six putative prophage regions in the SNUABM‐JN1 genome. Among them, PHAGE_Vibrio_12B12 located on Chromosome II (22,683 bp; 36 genes) was classified as intact and carried the ribosome‐associated toxin gene *ratA* (Table S6). IslandViewer 4 predicted genomic islands distributed across replicons, including 16 on Chromosome I, five on Chromosome II, two on plasmid II, and one on plasmid III (Table S7).

## 4. Discussion

Mass mortality accompanied by muscle whitening was observed for approximately 3 months in whiteleg shrimp farms located in regions adjacent to the west coast of the Republic of Korea. Molecular screening targeting major WOAH‐listed crustacean pathogens yielded negative results, prompting bacterial isolation from the whitened muscle tissue. In parallel, bacteria were isolated from the hepatopancreas, the principal target organ in shrimp diseases [41–43]. *P. damselae* subsp. *damselae* was predominantly isolated and ultimately confirmed to be subsp. *damselae* by 16S rRNA sequence comparison and *ureC*‐targeted PCR. The same subspecies was also isolated during the 2024 survey, whereas the 2025 survey suggested an increase in both the isolation frequency and geographic distribution. In this study, SNUABM‐JN1 was selected as a representative isolate for phenotypic and genomic characterization. Experimental infection confirmed that the resulting clinical signs were comparable to field observations.

Phenotypic findings suggested that SNUABM‐JN1 can tolerate various environmental conditions. The strain grew over a broad temperature range (10–40°C) and produced growth curves across a wide salinity range (0.5%–5.0% NaCl). These growth characteristics are consistent with previous descriptions of *P. damselae* subsp. *damselae* as a halophilic marine bacterium that requires NaCl for growth and can grow at NaCl concentrations ranging from 1% to 6%, with most subsp. *damselae* strains also capable of growth at 37°C [8, 17]. Notably, the lag phase was shortened at 29°C across all salinity conditions, suggesting that this strain may gain a growth advantage under elevated water temperature. This temperature‐associated physiological shift is consistent with previous observations that *P. damselae* subsp. *damselae* is repeatedly associated with disease outbreaks linked to increased water temperature [44]. Antimicrobial susceptibility testing showed that SNUABM‐JN1 was susceptible to all the evaluated antimicrobials. However, increasing antimicrobial resistance has been documented during certain disease outbreaks, underscoring the need for continued surveillance of antimicrobial susceptibility in aquaculture settings [45, 46]. Such surveillance efforts may also need to extend beyond cultured animals to the surrounding water, which is increasingly recognized as an important reservoir of antibiotics and antimicrobial resistance genes, as well as a site for the exchange of resistance genes between environmental and pathogenic bacteria [47].

In the challenge experiment, shrimp injected with 10^3^ to 10^5^ CFU⋅g^−1^ exhibited a lower survival rate at 29°C than at 25°C, and LD_50_ was also lower at 29°C. These findings align with the field observations, in which mortality increased sharply during periods of elevated water temperature. Collectively, these results suggest that higher temperatures may enhance bacterial virulence and compromise host defense in whiteleg shrimp. Temperature‐dependent upregulation of virulence factors has been reported in *P. damselae* subsp. *damselae* and other marine pathogens, such as *Vibrio coralliilyticus* [44, 48]. In addition, elevated temperatures have been associated with reduced immune indicators in whiteleg shrimp that contribute to the defense against pathogens [49]. Further studies tracking temperature‐dependent changes in SNUABM‐JN1 virulence gene expression, together with host immune parameters, would help clarify the mechanisms underlying the increased mortality observed at 29°C. Clinically, muscle whitening begins at the injection site and extends to the abdominal musculature, consistent with a recent report from Japan describing muscle whitening in kuruma shrimp infected with *P. damselae* subsp. *damselae* [18]. Histopathologically, hemocytic infiltration in the muscle was a common feature of both studies. However, the type of necrosis differed—liquefactive necrosis was reported in a Japanese study, whereas coagulative necrosis predominated in the present study.

The complete SNUABM‐JN1 genome comprised two circular chromosomes and four circular plasmids. Notably, the isolate lacked the pPHDD1 plasmid, which is associated with high virulence in *P. damselae* subsp. *damselae* [50]. pPHDD1 is known to carry hemolytic and cytotoxic toxin genes, such as phospholipase‐D damselysin (*Dly*) and the pore‐forming toxin phobalysin P (*PhlyP*), which contribute to host tissue damage and pathogenicity [51]. A kuruma shrimp isolate reported in Japan also lacked pPHDD1 yet remained pathogenic, suggesting that pPHDD1 may not be essential for shrimp pathogenicity in all contexts [18]. Therefore, comparative infection studies using pPHDD1‐positive and pPHDD1‐negative isolates would be informative for a more direct evaluation of the contribution of pPHDD1 to disease outcomes in shrimp. VFDB screening identified multiple motility‐related genes, including flagellar genes (*flhA*, *flhB*, *fliF*, *fliI*, and *fliM*) and chemotaxis‐related genes (*cheA* and *cheB*). Consistent with these genomic findings, TEM analysis revealed a single polar flagellum in SNUABM‐JN1, providing structural evidence for flagellum‐based motility. Together with the observed swimming motility phenotype, these findings support the presence of flagellum‐mediated motility in this strain.

The intact prophage PHAGE_Vibrio_12B12 identified on Chromosome II carried *ratA*, which encodes the ribosome association toxin RatA. In *Escherichia coli*, RatA has been reported to bind specifically to the 50S ribosomal subunit and prevent its association with the 30S subunit, thereby inhibiting 70S ribosome formation and translation initiation [52]. More broadly, genes encoded by prophages can influence bacterial fitness, stress resistance, biofilm formation, and virulence‐related traits, suggesting that prophages may contribute to bacterial adaptation beyond their role as genomic elements [53, 54]. In this context, the presence of *ratA* within an intact prophage represents an additional genomic feature of SNUABM‐JN1, although the expression and functional role of this gene during shrimp infection remain to be determined in future studies.

Although SNUABM‐JN1 was phenotypically susceptible to all 14 antimicrobials tested, the CARD analysis predicted antimicrobial resistance determinants associated with efflux pumps and target alterations. The presence of resistance genes does not necessarily confer a resistant phenotype under standard testing conditions, because such genes may remain weakly expressed or suppressed under baseline conditions [55]. Resistant phenotypes may emerge when gene expression is induced by regulatory changes, mutations, or environmental stressors [56]. Therefore, although the isolate appears broadly susceptible to standardized testing conditions, selective pressures commonly encountered in aquaculture environments, such as antimicrobial exposure, fluctuating water quality, and high stocking density, could increase the likelihood that latent resistance mechanisms are phenotypically expressed. Considering the risk of resistance selection under antimicrobial pressure and the limited adaptive immunity of shrimps, disease control in shrimp aquaculture often relies more heavily on prevention than on postoutbreak treatment, underscoring the need to develop alternatives to antibiotics [57, 58]. Bacteriophages are bacterial viruses that infect and lyse specific hosts and have been discussed as potential alternatives to antibiotics [59, 60]. Phage activity against *P. damselae* subsp. *damselae* has been reported [61, 62], and prophylactic phage application to whiteleg shrimp was effective in a previous study [63]. Probiotics have also been explored as a preventive approach, including pretreatment with rearing water and dietary supplementation, both of which have shown protective effects in shrimp aquaculture [64, 65].

## 5. Conclusions

In conclusion, the pathogenic *P. damselae* subsp. *damselae* was identified in whiteleg shrimp with muscle whitening, which is the first description of this infection in the Republic of Korea. Experimental challenge with strain SNUABM‐JN1 revealed temperature‐associated differences in shrimp survival, and histopathological examination revealed coagulative necrosis and hemocytic infiltration in both muscle and hepatopancreatic tissues. Phenotypic profiling, antimicrobial susceptibility testing, and whole genome sequencing collectively supported the characterization of the isolate and facilitated the interpretation of its virulence potential in the context of field outbreaks. These findings provide a basis for future studies on *P. damselae* subsp. *damselae* infection in shrimp, particularly to clarify how temperature shifts influence disease severity and to guide the development of prevention and control strategies. Beyond aquaculture, the capacity of *P. damselae* subsp. *damselae* to act as an opportunistic human pathogen warrants further investigation to clarify its environmental persistence, transmission routes, and potential public health implications.

## Author Contributions

**Jae Hong Park**: conceptualization, formal analysis, investigation, methodology, writing – original draft preparation. **Sib Sankar Giri**: validation, writing – review and editing. **Mae Hyun Hwang**: formal analysis, validation. **Da Sol Park**: data curation, visualization. **Eun Jae Park**: formal analysis, visualization. **Sung Jae Han**: data curation, resources. **Jin Woo Jun**: project administration, resources, supervision, writing – review and editing. **Se Chang Park**: funding acquisition, project administration, supervision, writing – review and editing.

## Funding

This study was supported by the Basic Science Research Program through the National Research Foundation of Korea (NRF) funded by the Ministry of Education (Grant RS‐2025‐16067617).

## Ethics Statement

Whiteleg shrimp (*Litopenaeus vannamei*) are invertebrates and are not covered under the Institutional Animal Care and Use Committee (IACUC) regulations of Seoul National University. Therefore, formal ethical approval and an approval ID were not required for this study.

## Conflicts of Interest

The authors declare no conflicts of interest.

## Supporting Information

Additional supporting information can be found online in the Supporting Information section.

## Supporting information


**Supporting Information** Table S1: Primer sequences and PCR conditions for the detection of major crustacean pathogens. Table S2: Primer sequences and RT‐PCR conditions for the detection of major crustacean pathogens. Figure S1: Differentiation of *P. damselae* subspecies by *ureC* gene detection. Lanes: M, 2000 bp DNA ladder; 1, SNUABM‐JN1; 2, SNUABM‐JB1; and 3, SNUABM‐CN1. Figure S2: Morphological characteristics of *P. damselae* subsp. *damselae* SNUABM‐JN1. (A) Colony morphology on tryptic soy agar supplemented with 1.5% NaCl (TSA+). (B) Colony morphology on thiosulfate citrate bile salts sucrose (TCBS) agar. (C) Gram‐stained cells observed under a light microscope (×1000). (D) Transmission electron micrograph of SNUABM‐JN1. Scale bar = 2.0 μm. Table S3: Antimicrobial susceptibility of *P. damselae* subsp. *damselae* SNUABM‐JN1. Table S4: Putative virulence–associated genes in *P. damselae* subsp. *damselae* SNUABM‐JN1. Table S5: Antimicrobial resistance determinants in *P. damselae* subsp. *damselae* SNUABM‐JN1. Table S6: Prophage regions predicted in *P. damselae* subsp. *damselae* SNUABM‐JN1. Table S7: Predicted genomic island regions in *P. damselae* subsp. *damselae* SNUABM‐JN1.

## Data Availability

The data supporting the findings of this study are available from the corresponding author upon reasonable request.
