# Supplementary material for: Characterization of Pathogenic Photobacterium damselae subsp. damselae From Whiteleg Shrimp (Litopenaeus vannamei): An Emerging Cause of Mass Mortality in the Republic of Korea
Source: Transbound Emerg Dis. 2026 Aug 6;2026:1555448. doi: 10.1155/tbed/1555448 (PMC13446146; doi:10.1155/tbed/1555448)
Supplement: Supplementary file 1 [file TBED-2026-1555448-s001.docx]

**TABLE S1**: Primer sequences and PCR conditions for the detection of major crustacean pathogens.

| **Target pathogen** | **Primer** | **Sequence (5'–3')** | **No. of Cycles** | **Temperature** | **Duration** | **Amplicon size (bp)** | **Reference** |
| --- | --- | --- | --- | --- | --- | --- | --- |
| AHPND | VpPirA-284F | TGACTATTCTCACGATTGGACTG | 1X  35X  1X | 94°C  94°C 60°C 72°C  72°C | 3 min  30 s 30 s 30 s  7 min | 284 | [23] |
|  | VpPirA-284R | CACGACTAGCGCCATTGTTA |  |  |  |  |  |
|  | VpPirB-392F | TGATGAAGTGATGGGTGCTC |  |  |  | 392 |  |
|  | VpPirB-392R | GTAAGCGCCGTTTAACTCA |  |  |  |  |  |
| DIV1 | CQIV-MCP-F | GAAACTTTATGCACAATCTTAT | 25X | 94°C 55°C 72°C | 30 s 30 s 30 s | 103 | [27] |
|  | CQIV-MCP-R | CCAATCATGTTGTCGTATCC |  |  |  |  |  |
| *Hepatobacter penaei* | NHPF2 | CGTTGGAGGTTCGTCCTTCAGT | 35X | 95°C 60°C 72°C | 30 s 30 s 30 s | 379 | [22] |
|  | NHPR2 | GCCATGAGGACCTGACATCATC |  |  |  |  |  |
| IHHNV | 309F | TCCAACACTTAGTCAAAACCAA | 35X | 94°C 55°C 72°C | 30 s 30 s 30 s | 309 | [26] |
|  | 309R | TGTCTGCTACGATGATTATCCA |  |  |  |  |  |
| WSSV (Primary) | 146F1 | ACTACTAACTTCAGCCTATCTAG | 39X | 94°C 55°C 72°C | 1 min 1 min 2 min | 1447 | [24] |
|  | 146R1 | TAATGCGGGTGTAATGTTCTTACGA |  |  |  |  |  |
| WSSV (Nested) | 146F2 | GTAACTGCCCCTTCCATCTCCA | 39X | 94°C 55°C 72°C | 1 min 1 min 2 min | 941 |  |
|  | 146R2 | TACGGCAGCTGCTGCACCTTG |  |  |  |  |  |
| EHP | EHP-510F | GCCTGAGAGATGGCTCCCACGT | 1X 35X   1X | 94°C  94°C  60°C  72°C  72°C | 3 min  30 s 30 s 30 s 5 min | 510 | [25] |
|  | EHP-510R | GCGTACTATCCCCAGAGCCCGA |  |  |  |  |  |

**TABLE S2**: Primer sequences and RT-PCR conditions for the detection of major crustacean pathogens.

| **Target pathogen** | **Primer** | **Sequence (5'–3')** | **No. of Cycles** | **Temperature** | **Duration** | **Amplicon size (bp)** | **Reference** |
| --- | --- | --- | --- | --- | --- | --- | --- |
| IMNV (Primary) | 4587F | CGACGCTGCTAACCATACAA | 45X  1X | 95°C  60°C 60°C | 45 s  45 s 7 min | 328 | [29] |
|  | 4914R | ACTCGGCTGTTCGATCAAGT |  |  |  |  |  |
| IMNV (Nested) | 4725 NF | GGCACATGCTCAGAGACA | 39X   1X | 95°C 65°C 72°C 72°C | 30 s 30 s 30 s 2 min | 139 |  |
|  | 4863 NR | AGCGCTGAGTCCAGTCTTG |  |  |  |  |  |
| MrNV | MrNV-F | GATACAGATCCACTAGATGACC | 1X 30X   1X | 95°C 94°C 55°C 68°C 68°C | 2 min 40 s 40 s 1 min 10 min | 681 | [31] |
|  | MrNV-R | GACGATAGCTCTGATAATCC |  |  |  |  |  |
| TSV | 9992 | AAGTAGACAGCCGCGCTT | 40X | 94°C 60°C | 45 s 45 s | 231 | [28] |
|  | 9195R | TCAATGAGAGCTTGGTCC |  |  |  |  |  |
| YHV1 | 10F | CCGCTAATTTCAAAAACTACG | 40X   1X | 94°C  58°C  68°C  68°C | 30 s  45 s 45 s 7 min | 135 | [30] |
|  | 144R | AAGGTGTTATGTCGAGGAAGT |  |  |  |  |  |

**FIGURE S1**: Differentiation of *P. damselae* subspecies by *ureC* gene detection. Lanes: M, 2,000bp DNA ladder; 1, SNUABM-JN1; 2, SNUABM-JB1; 3, SNUABM-CN1.

**FIGURE S2**: Morphological characteristics of *P. damselae* subsp. *damselae* SNUABM-JN1. (A) Colony morphology on tryptic soy agar supplemented with 1.5% NaCl (TSA+). (B) Colony morphology on thiosulfate citrate bile salts sucrose (TCBS) agar. (C) Gram-stained cells observed under a light microscope (×1,000). (D) Transmission electron micrograph of SNUABM-JN1. Scale bar = 2.0 μm.

**TABLE S3**: Antimicrobial susceptibility of *P. damselae* subsp. *damselae* SNUABM-JN1.

| **Antimicrobial agents** | **Zone diameter (mm, mean ± SD)** | **Sensitivity** |
| --- | --- | --- |
| Ampicillin | 23.57 ± 0.72 | S |
| Ampicillin-sulbactam | 29.05 ± 0.35 | S |
| Cefepime | 27.95 ± 0.41 | S |
| Cefotaxime | 32.32 ± 0.46 | S |
| Cefoxitin | 32.35 ± 1.02 | S |
| Ceftazidime | 28.91 ± 0.06 | S |
| Imipenem | 30.63 ± 0.21 | S |
| Meropenem | 23.51 ± 0.35 | S |
| Amikacin | 22.23 ± 0.65 | S |
| Gentamicin | 21.85 ± 1.01 | S |
| Tetracycline | 32.72 ± 0.12 | S |
| Ciprofloxacin | 40.05 ± 1.38 | S |
| Ofloxacin | 40.71 ± 0.22 | S |
| Chloramphenicol | 35.33 ± 0.55 | S |

**TABLE S4**: Putative virulence-associated genes in *P. damselae* subsp. *damselae* SNUABM-JN1.

| **Category** | **Virulence factor** | **Related genes** |
| --- | --- | --- |
| Adherence | ChiRP | *pilB, pilD/vcpD* |
|  | EF-Tu | *tufA* |
|  | Hsp60 | *htpB* |
|  | IlpA | *IlpA* |
|  | MSHA pili | *mshE* |
|  | Tap type IV pili | *tapT* |
|  | Type IV pili | *vfr* |
| Antimicrobial activity /Competitive advantage | AcrAB | *acrB* |
| Biofilm | AI-2 | *luxS* |
|  | Alginate | *algU* |
| Effector delivery system | T4SS secreted effectors | *CBU_1566* |
|  | T6SS | *hcp1* |
|  | VAS T6SS | *clpB/vasG, icmF/vasK, vasA, vasB, vasE, vasF, vgrG-2, vipB/mglB* |
| Immune modulation | Capsule | *rpe, ugd* |
|  | LOS | *galE, galU, gmhA/lpcA, kdsA, lpxB, lpxC, lpxD, msbA, rffG* |
|  | LPS | *wbtL* |
| Motility | Flagella | *cheA, cheB, fleR/flrC, flgP, flhA, flhB, fliF, fliI, fliM, flrA* |
|  | Polar flagella | *flmH, nueA* |
| Nutritional /Metabolic factor | Acinetobactin | *basG* |
| Others | Isocitrate lyase | *icl* |
| Regulation | RpoS | *rpoS* |
| Stress survival | ClpP | *clpP* |
|  | KatAB | *katA* |
|  | Urease | *ureB, ureG* |

**TABLE S5**: Antimicrobial resistance determinants in *P. damselae* subsp. *damselae* SNUABM-JN1.

| **Location** | **ARO Term** | **Drug class** | **Resistance mechanism** |
| --- | --- | --- | --- |
| Chromosome I | qacJ | disinfecting agents and antiseptics | antibiotic efflux |
|  | adeF | fluoroquinolone antibiotic, tetracycline antibiotic | antibiotic efflux |
|  | CRP | macrolide antibiotic, fluoroquinolone antibiotic, penicillin beta-lactam | antibiotic efflux |
|  | Escherichia coli EF-Tu mutants conferring resistance to Pulvomycin | elfamycin antibiotic | antibiotic target alteration |
|  | Haemophilus influenzae PBP3 conferring resistance to beta-lactam antibiotics | cephalosporin, penicillin beta-lactam | antibiotic target alteration |
|  | Escherichia coli EF-Tu mutants conferring resistance to Pulvomycin | elfamycin antibiotic | antibiotic target alteration |
| Chromosome II | adeF | fluoroquinolone antibiotic, tetracycline antibiotic | antibiotic efflux |

**TABLE S6**: Prophage regions predicted in *P. damselae* subsp. *damselae* SNUABM-JN1.

| **Prophage** | **Location** | **Completeness** | **Region position** |
| --- | --- | --- | --- |
| PHAGE_Vibrio_12B12 | Chromosome I | Incomplete | 1960-18322 |
| PHAGE_Klebsi_ST13_OXA48phi12.1 |  | Incomplete | 493562-505459 |
| PHAGE_Escher_500465_1 |  | Incomplete | 741481-772186 |
| PHAGE_Vibrio_12B12 | Chromosome II | Intact | 293558-316241 |
| PHAGE_Escher_RCS47 | plasmid I | Incomplete | 2523-17078 |
| PHAGE_Salmon_SJ46 |  | Incomplete | 35840-85052 |

**TABLE S7**: Predicted genomic island regions in *P. damselae* subsp. *damselae* SNUABM-JN1.

| **Location** | **Start** | **End** | **Size (bp)** | **No. of CDS** |
| --- | --- | --- | --- | --- |
| Chromosome I | 940 | 7,783 | 6,843 | 15 |
|  | 383,225 | 387,259 | 4,034 | 3 |
|  | 452,398 | 459,854 | 7,456 | 10 |
|  | 461,977 | 469,406 | 7,429 | 5 |
|  | 487,176 | 500,279 | 13,103 | 13 |
|  | 702,203 | 715,738 | 13,535 | 5 |
|  | 746,929 | 759,115 | 12,186 | 13 |
|  | 748,044 | 755,128 | 7,084 | 9 |
|  | 1,207,360 | 1,215,684 | 8,324 | 9 |
|  | 1,776,152 | 1,788,365 | 12,213 | 21 |
|  | 2,461,762 | 2,473,671 | 11,909 | 14 |
|  | 2,487,995 | 2,496,004 | 8,009 | 9 |
|  | 2,603,604 | 2,608,507 | 4,903 | 3 |
|  | 2,781,531 | 2,796,951 | 15,420 | 10 |
|  | 3,275,271 | 3,286,875 | 11,604 | 14 |
|  | 3,276,707 | 3,288,472 | 11,765 | 14 |
| Chromosome II | 288,886 | 309,060 | 20,174 | 30 |
|  | 300,898 | 347,847 | 46,949 | 36 |
|  | 384,936 | 423,142 | 38,206 | 32 |
|  | 1,261,865 | 1,285,011 | 23,146 | 20 |
|  | 1,274,037 | 1,278,073 | 4,036 | 4 |
| plasmid II | 49,855 | 57,392 | 7,537 | 9 |
|  | 73,000 | 79,383 | 6,383 | 6 |
| plasmid III | 19,882 | 26,831 | 6,949 | 9 |
